# Supplementary material for: Sensory event-related potential morphology predicts age in premature infants
Source: Clin Neurophysiol. Author manuscript; Available in PMC 2025 Sep 17. (PMC7618130; doi:10.1016/j.clinph.2023.11.007)
Supplement: Supplementary Material [file EMS206629-supplement-Supplementary_Material.docx]

***Supplementary Material:***

***Sensory event-related potential morphology predicts age in premature infants***

Coen S. Zandvoort, Marianne van der Vaart, Shellie Robinson, Fatima Usman, Gabriela Schmidt Mellado, Ria Evans Fry, Alan Worley, Eleri Adams, Rebeccah Slater, Luke Baxter, Maarten de Vos, Caroline Hartley

***Supplementary Results***

*Visual and tactile models*

Besides the brain age model that was constructed from both visual and tactile magnitudes, we also made single-modality models using either visual or tactile magnitudes as input. For the training set, mean absolute errors were higher compared to the visual-tactile model but age prediction performance was still significantly different from the average age model (visual only: mean absolute error: 1.77 weeks with 95% at [1.46; 2.16], *p* = 0.0003; Figure S6, tactile only: mean absolute error: 1.67 weeks with 95% at [1.35; 2.03], *p* = 0.0024; Figure S7). However, in the independent test sample, the single-stimulus models of visual and tactile responses, age prediction was not significantly different from the average age model (visual - mean absolute errors = 1.75 weeks with 95% at [1.51, 2.03], *p* = 0.0001; Figure S6; tactile mean absolute error = 1.77 weeks with 95% at [1.44; 2.14], *p* = 0.0005; Figure S7).

*Deviations in sensory development may be predictive of later life neurodevelopmental abnormalities – exploratory pilot data 2*

To exclude that the results as presented in Figure 6 were related to bias in our model (particularly at older ages, where the error in the training set is greater than for infants at approximately 34 weeks and the brain age may be underpredicted, Figure 4a), we recalculated the gradients without recordings from when the infants were older than 37 weeks. Restricting the age range, the mean gradients for the two infants with below average Bayley’s outcomes equalled 0.48 and for the three infants with average Bayley’s outcomes equalled 0.79. Secondly, we estimated the bias in the training set and removed this from the test set (Figure S8). After bias removal, whilst the gradients of the brain age trajectories were closer to 1 (gradient of 1.00 for infants with below average Bayley’s and 1.37 for those with average outcomes, Figure S8) but the mean absolute error was still higher in the infants with below average outcome (1.91 compared with 1.57 weeks), supporting the finding that brain age may be indicative of later life outcome.***Supplementary figures***


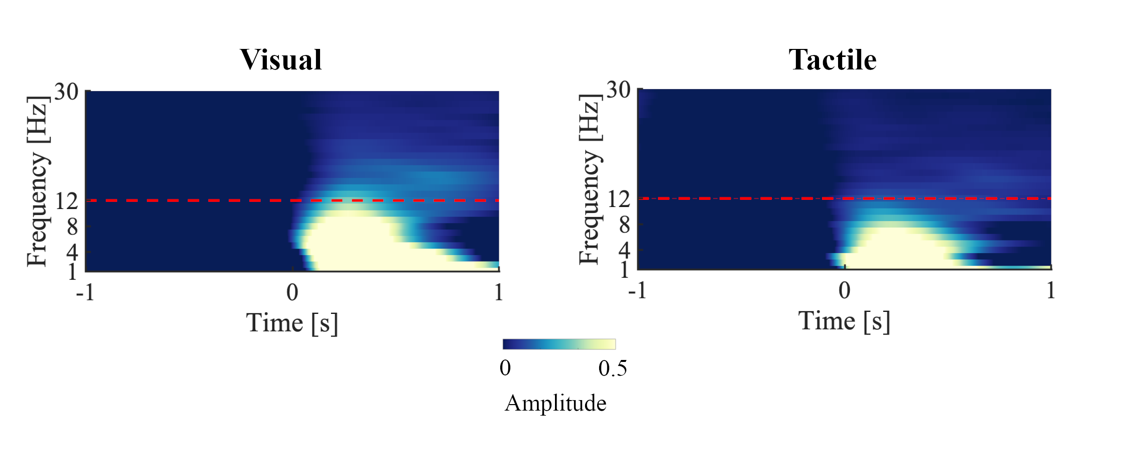


Figure S1. Time-frequency amplitudes of the visual- and tactile-evoked potentials of the training sample. Each time-locked evoked response was bandpass filtered between 1 and 30 Hz with cut-off frequencies of +/- 1 Hz around the frequency of interest. Hilbert transforms of the bandpass filtered signals and its instantaneous amplitude was computed by taking the modulus. Time at 0 sec indicates stimulus onset. Horizontal dashed red line corresponds to the upper frequency cut-off used for NRF computation.

*
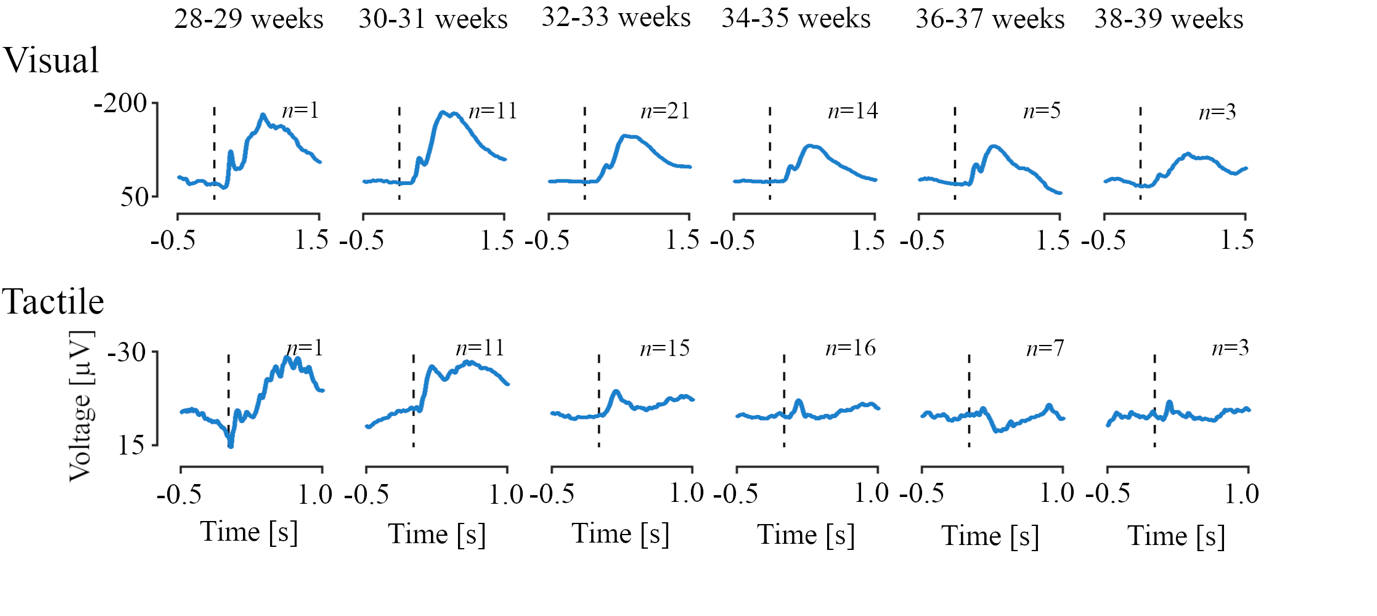
*

Figure S2. Stimulus-evoked electroencephalographic potentials according to infant age. Age-dependent evoked potentials for two-weeks intervals between 28 to 40 weeks of post-menstrual age for the visual and tactile stimuli at channels Oz and Cz, respectively, for the test set (see Figure 2 for the training set). Woody filtering aligned the responses to their age-weighted averages. Vertical dashed lines correspond to the stimulus onset.

*
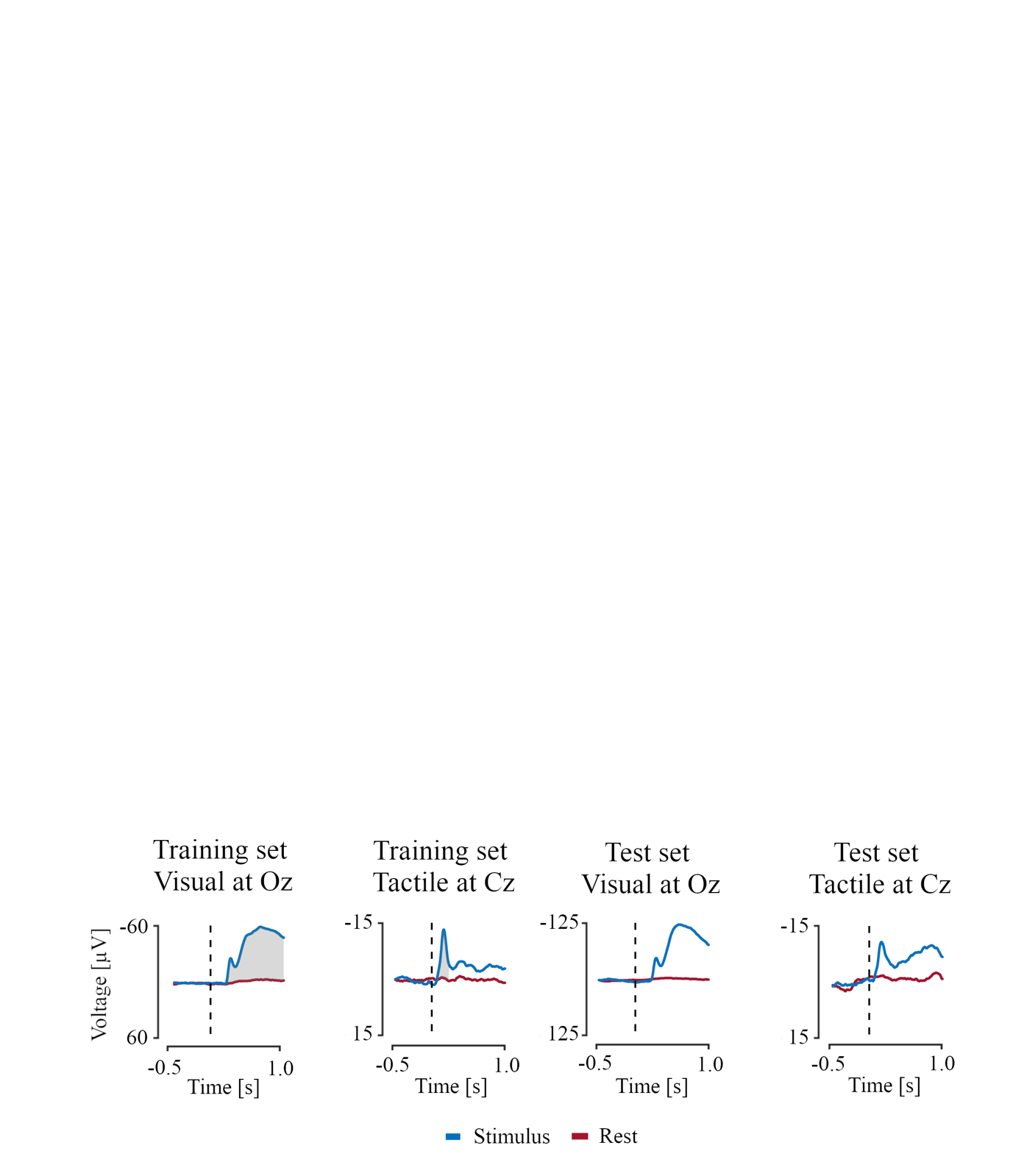
*

*Figure S3. Grand average means of the stimulus response (in blue) and resting state (in red). Grey areas depict the time windows where the stimulus and resting state means are significantly different as identified by the cluster-based permutation testing. This was only applied to the responses of the training set.*


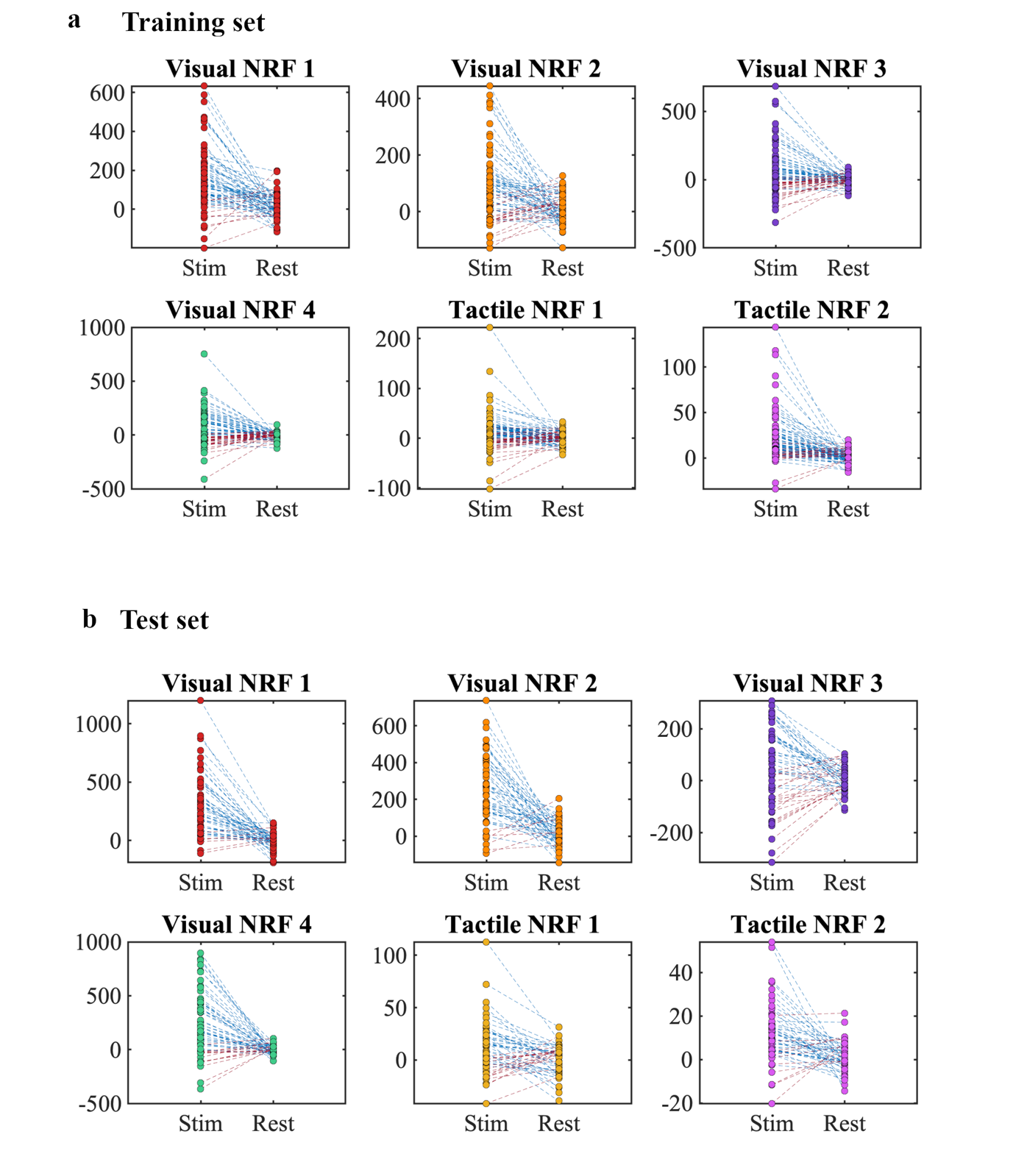


*Figure S4.* ***a)*** *Training and* ***b)*** *test set magnitudes for stimulus-evoked potentials (Stim) and resting state activity (Rest) using the six neurodynamic response functions (NRFs) identified in the training set. Magnitudes were estimated for the stimulus responses and resting state activity of each recording. Dashed lines connect the two magnitudes of each recording, where blue means a higher magnitude in the stimulus condition relative to the resting state condition and red a lower magnitude for the stimulus condition.*


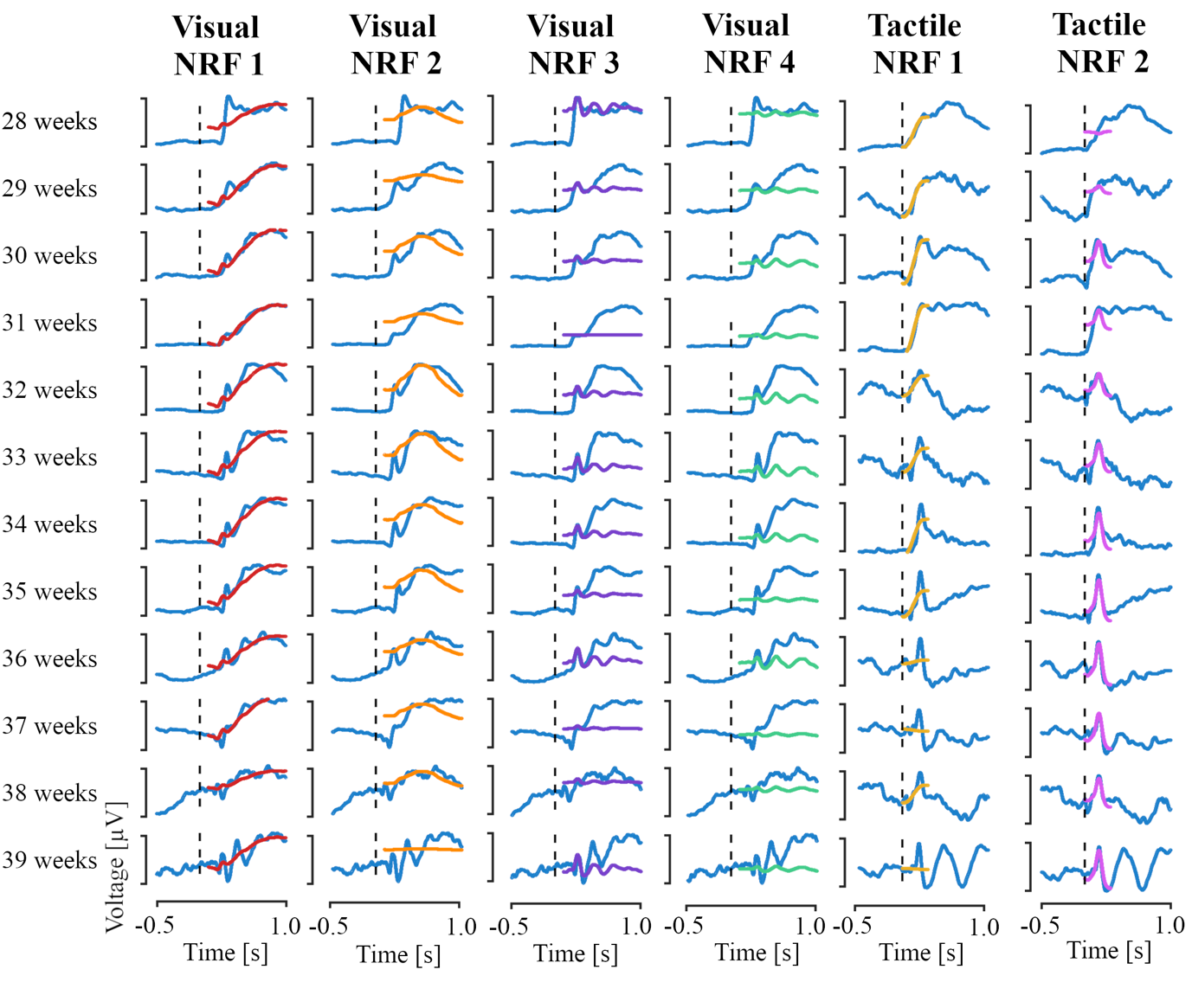


*Figure S5. Age-dependent neurodynamic response function (NRF) projections of the visual and tactile NRFs on the training set responses. Each individual projection is plotted on its own individual y-scale. NRFs were projected on age averages after computing age-weighted evoked potentials using linear regression models (see methods). These age-weighted potentials were Woody filtered to the NRF after which the NRF was projected on the EEG traces. Vertical dashed lines correspond to time = 0 seconds (i.e., the stimulus onset).*

*
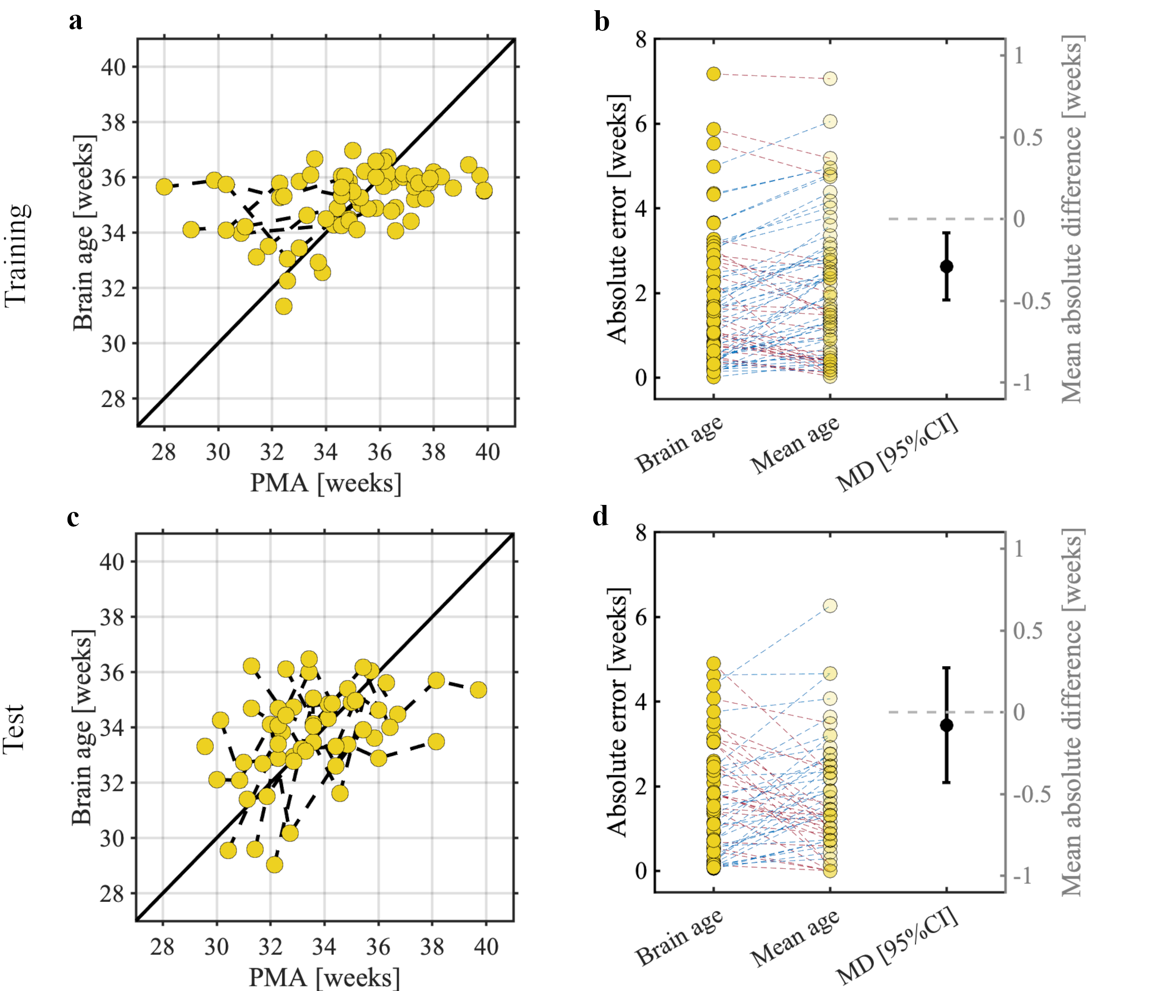
*

*Figure S6. Brain age prediction models and their statistical evaluations for the* ***a-b)*** *training and* ***c-d)*** *test samples. Panels a and c show the post-menstrual age (PMA) and brain age using leave-one-infant-out cross-validation. Predictions are based on the visual model. Each dot indicates a single recording with PMA predicted using the stimulus responses. Dashed black lines between dots are infants that took part in multiple recordings. Solid black line indicates perfect prediction. Panels b and d depict the comparison in absolute errors between the Brain age and null model (Mean age) and its mean (absolute) difference including 95% confidence interval (i.e., MD [95%CI]). Blue dashed lines mean a higher absolute error for the mean age prediction relative to the brain age prediction, and red yield a lower absolute error for the mean age.*

*
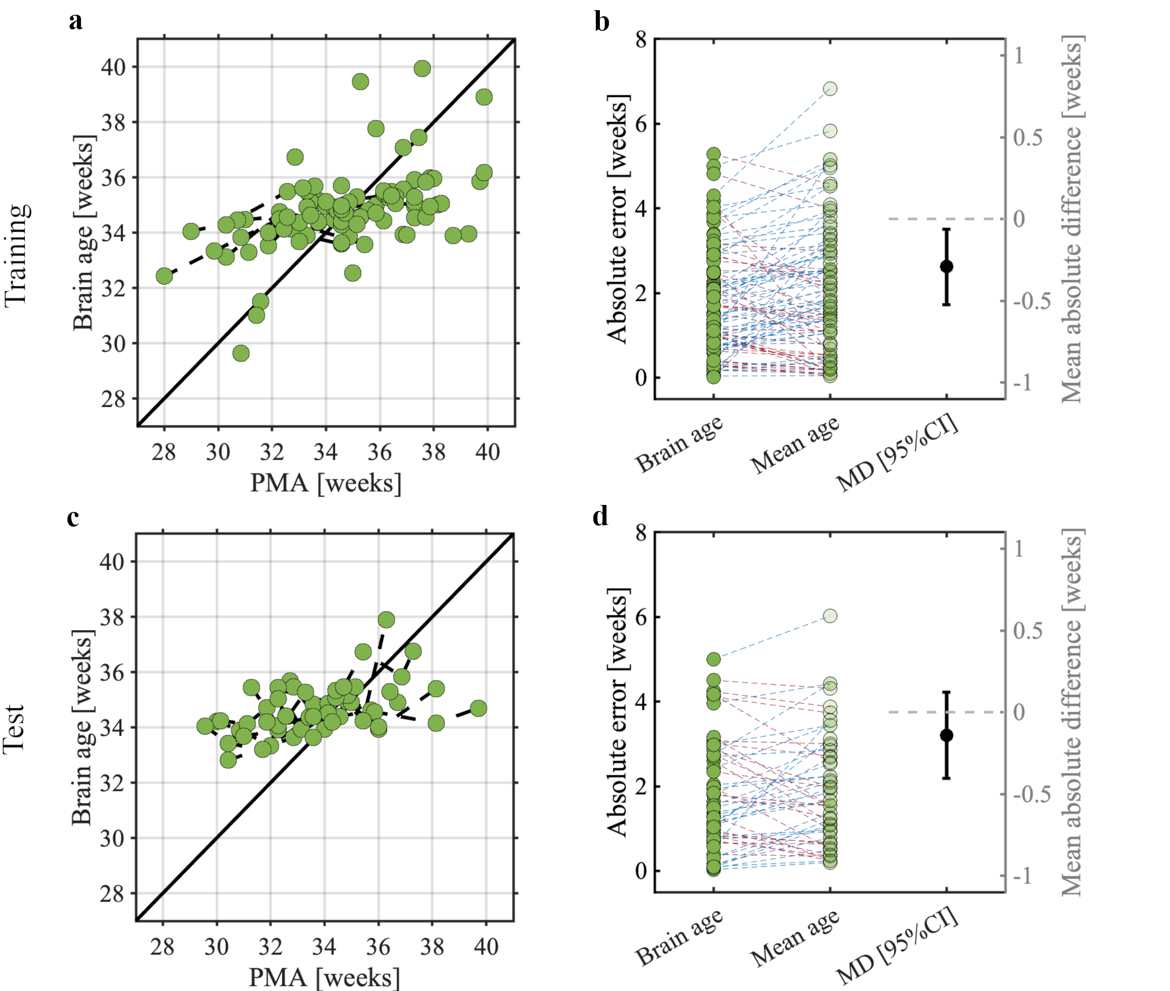
*

*Figure S7. Brain age prediction models and their statistical evaluations for the* ***a-b)*** *training and* ***c-d)*** *test samples. Panels a and c show the post-menstrual age (PMA) and brain age using leave-one-infant-out cross-validation. Predictions are based on the tactile model. Each dot indicates a single recording with PMA predicted using the stimulus responses. Dashed black lines between dots are infants that took part in multiple recordings. Solid black line indicates perfect prediction. Panels b and d depict the comparison in absolute errors between the Brain age and null model (Mean age) and its mean (absolute) difference including 95% confidence interval (i.e., MD [95%CI]). Blue dashed lines mean a higher absolute error for the mean age prediction relative to the brain age prediction, and red yield a lower absolute error for the mean age.*


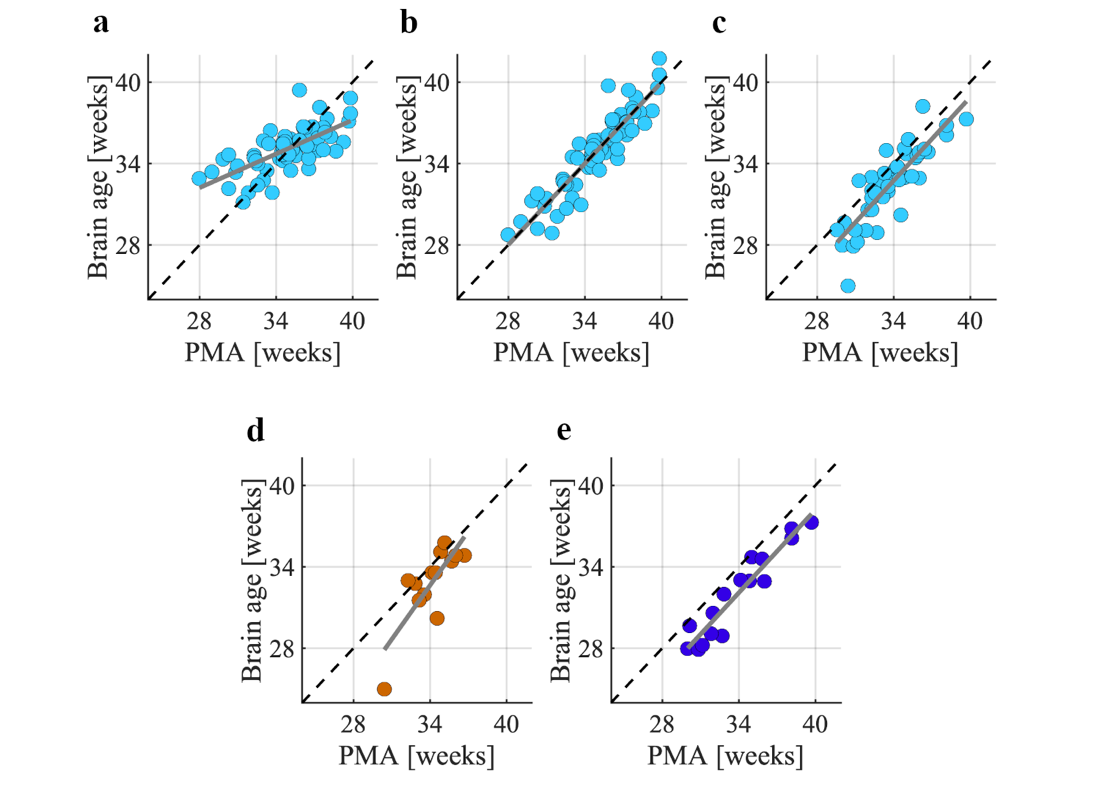


Figure S8. Brain age predictions from which the bias (i.e., deviations from perfect predictions) are removed. Model bias estimated in the **a)** training set by fitting a line of best fit between the brain age and post-menstrual (PMA) data as solid grey graph. Bias was estimated by taking the difference between this line of best fit and the perfect predictions, and subsequently removed from the brain age predictions of the training set, with the resulting brain age predictions shown in panel **b**) Brain age predictions of younger and older infants are particularly adjusted after bias removal. The line of best fit/bias estimated in the training set was used to remove bias in **c)** the test set, **d)** infants in the test set with average Bayley’s outcomes, and **e)** infants in the test set with below average Bayley’s outcomes following bias removal.
